# Mendelian randomization as an approach to assess causal effects of inflammatory bowel disease on atrial fibrillation

**DOI:** 10.18632/aging.202906

**Published:** 2021-04-06

**Authors:** LaiTe Chen, GuoSheng Fu, ChenYang Jiang

**Affiliations:** 1 Department of Cardiology of Sir Run Run Shaw Hospital, School of Medicine, Zhejiang University, HangZhou, China

**Keywords:** atrial fibrillation, inflammatory bowel disease, mendelian randomization, single nucleotide polymorphisms, risk factor

## Abstract

Background: Despite growing evidence indicating that patients with inflammatory bowel disease (IBD) have an increased risk of atrial fibrillation (AF), owing to the potential biases of confounding effects and reverse causation, the specific relationship between IBD and AF remains controversial. The aim of this study is to determine whether there is a causal effect of IBD on AF.

Methods: A two-sample Mendelian randomization (MR) study was performed to evaluate the causal effect of IBD on AF. Statistical summaries for the associations between single nucleotide polymorphisms (SNPs) and traits of interest were obtained from independent consortia with European populations. The dataset of IBD was acquired from genome-wide association studies (GWAS), including more than 75,000 cases and controls. A GWAS with 60,620 AF cases and 970,216 controls was used to identify genetic variation underlying AF. The causal effect was estimated using the multiplicative random effects inverse-variance weighted method (IVW), followed by sensitivity analysis.

Results: Using 81 SNPs, there was no evidence to suggest an association between genetically predicted IBD and risk of AF with multiplicative random-effects IVW MR analysis (odds ratio = 1.0000, 95% confidence interval: 0.9994 1.0005, p = 0.88).

Conclusion: As opposed to current assumptions, no substantial evidence was found to support a causal role of IBD in the development of AF.

## INTRODUCTION

Atrial fibrillation (AF) is the most common type of arrhythmia that causes hemodynamic disorders and thrombotic strokes. Important etiologies of AF include surgical procedures [1], hyperthyroidism [2], myocardial infarction [3], and cardiomyopathies [4]. Although the potential pathophysiologic mechanisms of AF remain controversial, there has been increasing evidence that inflammation is involved in the pathogenesis of AF [5–7]. Laboratory tests showed that inflammatory biomarkers (high-sensitivity C-reactive protein) increased in patients with AF [8] and anti-inflammatory medications may reduce the morbidity of AF [9].

Characterized by chronic diarrhea, abdominal pain, and perianal bleeding, inflammatory bowel disease (IBD) is rising throughout the world. IBD severely impacts all aspects of life while increasing the burden on health care [10]. IBD, commonly categorized as a relapsing idiopathic inflammatory disease of ulcerative colitis (UC) and Crohn’s disease (CD), is an immune-mediated chronic inflammatory disorder of the gastrointestinal tract [11]. Several recent studies have suggested a positive association between IBD and AF [12–14]. Various pathological processes such as oxidative stress, fibrosis, and apoptosis, are involved in the systemic inflammation of IBD and lead to structural and electrical remodeling of the atria, which may contribute to the development of AF [15]. Despite growing evidence indicating that patients with IBD have an increased risk of AF, owing to the potential biases of confounding effects and reverse causation [16], the specific relationship between IBD and AF remains controversial.

Mendelian randomization (MR) analysis exploits genetic variants as instrumental variables to establish a strong causal inference between exposure and risk of disease without involving potential confounders and reverse causation [17]. In this study, MR analysis was applied to evaluate the causal association between IBD and AF.

## RESULTS

**Figure 1:**
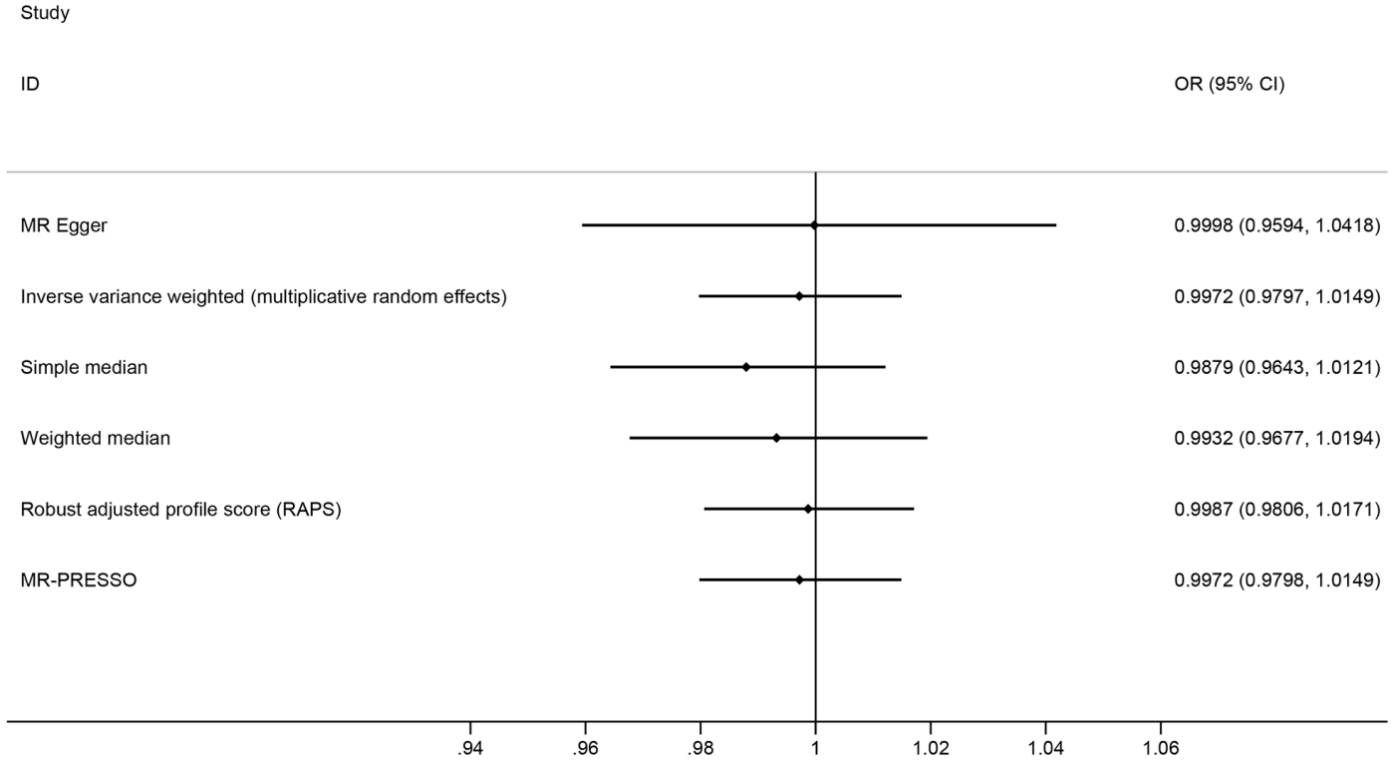
Mendelian randomization estimates of the causal effect of inflammatory bowel disease on atrial fibrillation.

**Figure 2:**
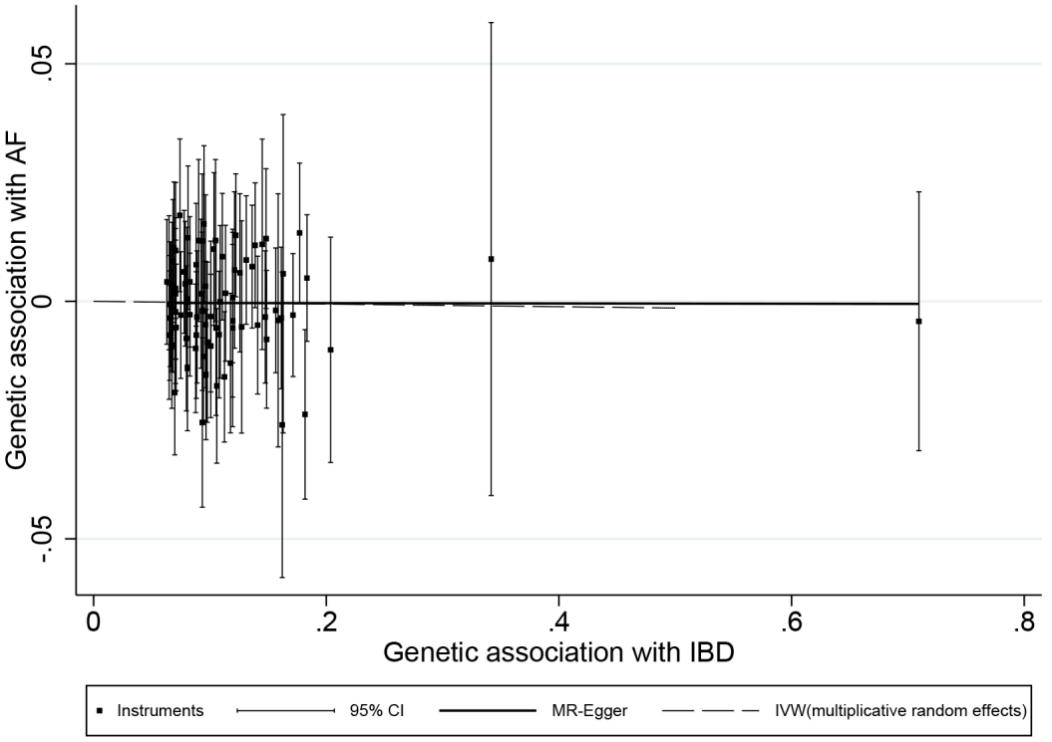
**Scatter plot of genetic associations with atrial fibrillation against associations with inflammatory bowel disease, with causal estimates (β coefficients) of inflammatory bowel disease on atrial fibrillation estimated by inverse-variance weighted (dashed line), and MR-Egger (solid line) methods.** The straight lines should be the change in the log odds of atrial fibrillation per unit increase of the log odds of inflammatory bowel disease.

There is a lack of evidence to suggest an association between genetic predisposition to IBD and AF (Supplementary Table 1). As the primary estimator, the multiplicative random effect IVW model showed that genetic predisposition to IBD was not associated with the risk of AF (OR = 0.9972, 95% CI: 0.9797 1.0149, p = 0.75, Figures 1, 2). A null association was also observed using the MR-Egger (OR = 0.9998, 95% CI: 0.9594 1.0418, p = 0.99, Figures 1, 2), simple median (OR = 0.9879, 95% CI: 0.9643 1.0121, p = 0.33, Figure 1), weighted median (OR = 0.9932, 95% CI: 0.9677 1.0194, p = 0.61, Figure 1), RAPS (OR = 0.9987, 95% CI: 0.9806 1.0171, p = 0.89, Figure 1) and MR-PRESSO methods (OR = 0.9972, 95% CI: 0.9798 1.0149, p = 0.76, Figure 1). Causal estimates of each SNP are listed in Supplementary Table 1.

**Figure 3:**
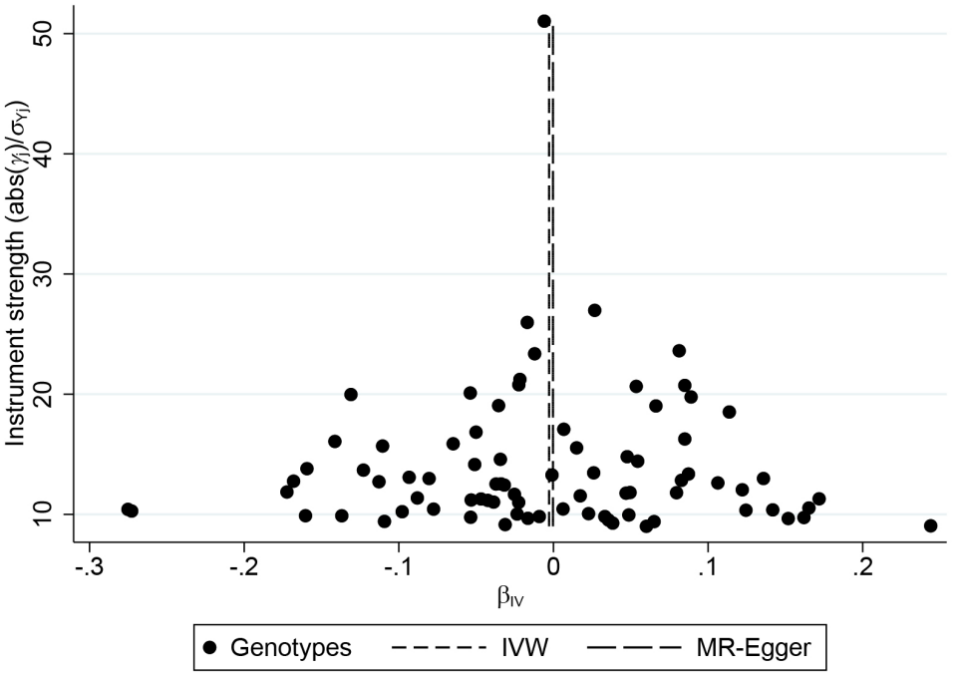
**Funnel plot of genetic associations with inflammatory bowel disease against causal estimates based on each genetic variant individually, where the causal effect is expressed in logs odds ratio of atrial fibrillation for each unit increase in inflammatory bowel disease.** The overall causal estimates (β coefficients) of inflammatory bowel disease on atrial fibrillation estimated by inverse-variance weighted (short dash line) and MR-Egger (long dash line) methods are shown.

**Figure 4:**
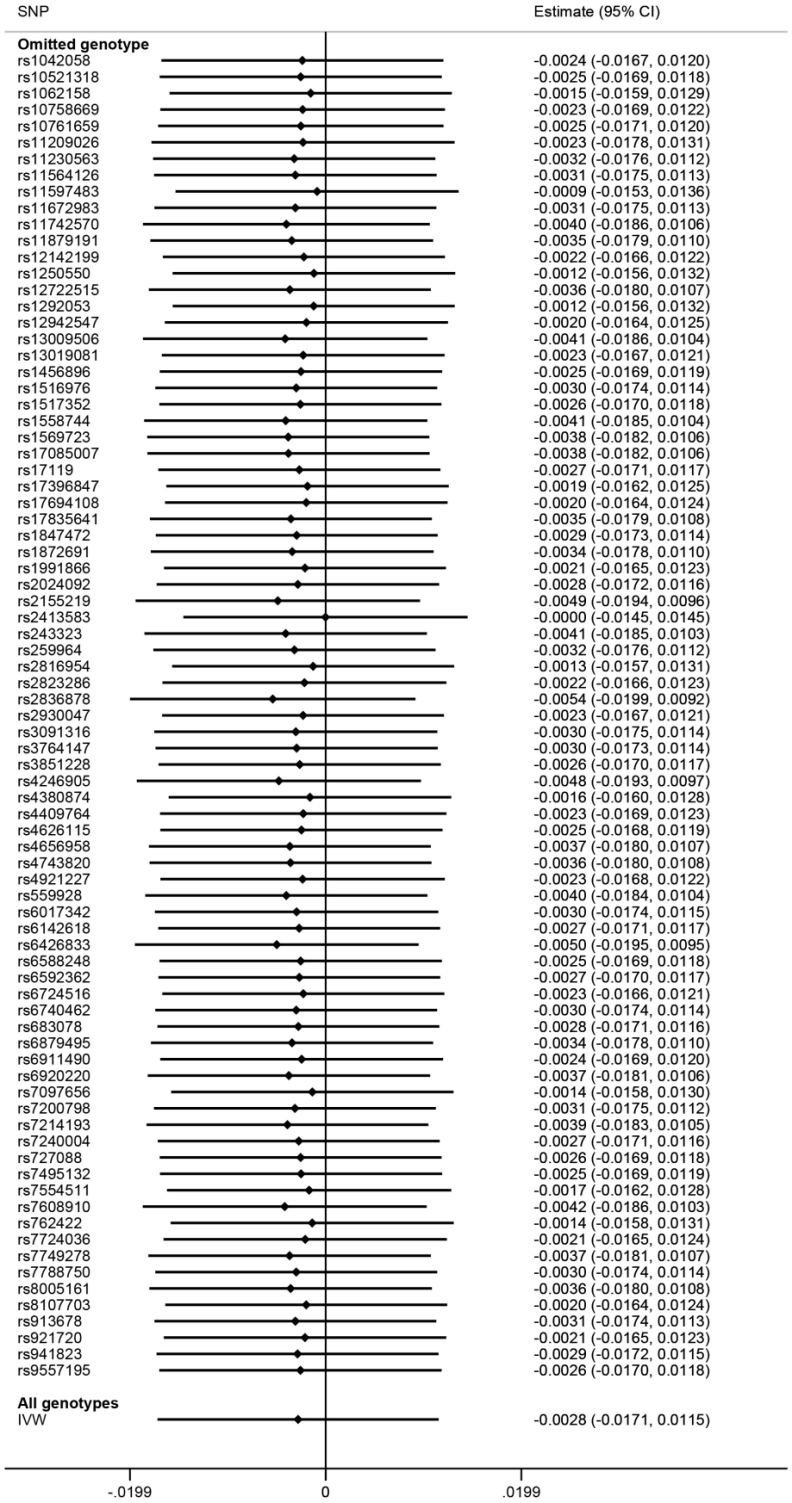
**Mendelian randomization leave-one-out sensitivity analysis for inflammatory bowel disease on atrial fibrillation.** Estimate is indicated by Odds Ratio (OR). SNP, Single Nucleotide Polymorphisms; CI, Confidence Interval; IVW, Inverse-Variance Weighted.

There was no evidence of substantial heterogeneity in the IVW analysis (Q = 117.1502, p = 0.0028, I^2^ = 0.3342), and the MR-PRESSO global test of heterogeneity also demonstrated the same result, after removing rs10800309, rs2266959, and rs12946510 for heterogeneity (p = 0.76). MR-Egger regression showed no evidence of directional pleiotropy for the association between the included SNP and the risk of AF (intercept = -0.0003, 95% CI: -0.0052 0.0045, p = 0.89). The funnel plot also showed no evidence of obvious heterogeneity across the estimates (Figure 3). The results of leave-one-out sensitivity analysis showed that the null association between genetic predisposition to IBD and AF was not remarkably affected by any individual SNP (Figure 4).

## DISCUSSION

Using two-sample MR analysis based on datasets from large-scale GWAS studies, our study demonstrated that genetic predisposition to IBD was not associated with the risk of AF. The findings were robust in sensitivity analyses with different instruments and statistical models. The datasets used for both SNP IBD and SNP AF estimates were acquired from European studies with a similar population, which minimized the possibility of population stratification bias. Using both the GWAS and the Immunochip associations, SNP IBD associations were estimated in a combined analysis, comprising 20,700 Crohn’s disease, 17,865 ulcerative colitis, and 37,747 healthy controls [18]. A total of 34,740,186 genetic variants from six contributing studies of European ancestry were tested to estimate SNP AF associations, identifying 111 genomic regions with at least 1 genetic variant associated with AF (P < 5 × 10^−8^) [19].

Several studies have shown that patients with IBD have a higher risk of developing AF [15, 20], especially during the active stage of IBD [13]. Using a population-based cohort study, You-Jung Choi and his colleagues identified 1,120 AF cases. Multivariable Cox regression indicated that patients with IBD had a 36% higher risk of AF than controls [15]. A systematic review and meta-analysis suggested that as compared to controls, IBD patients were at a 2.2-fold increased risk of developing AF [20]. This increased risk of AF in IBD, although not entirely understood, has been proposed to have different underlying pathophysiologies. Myocardial inflammation and fibrosis seem to be the pivotal factors [21]. Inflammatory processes and oxidative stress lead to cardiomyocyte necrosis, with subsequent electrical and structural remodeling [22]. Other than idiopathic electrophysiological abnormalities, left atrial volume and mechanical function degeneration were detected using echocardiography in patients with UC, indicating that structural changes also could lead to the development of AF [23].

In this study, MR analysis was performed as it can control potential confounders and avoid reverse causation. MR analysis did not provide sufficient evidence to support a positive causal effect of IBD on the risk of AF. The lack of a genetic causal effect of IBD with the risk of AF suggested that the positive linkage between the presence of IBD and the risk of developing AF demonstrated in previous observational studies may have been the residual confounding due to common risk factors. IBD and AF share several lifestyle modifiable risk factors. Increased risk for IBD was found in patients with habits of smoking and alcohol intake [24, 25]. On the other hand, it is well established that alcohol not only does not extend cardioprotection to AF but is also an important risk factor for AF [26]. A systematic review also showed that high alcohol intake was associated with an increased incidence of AF [27]. In addition, as an immunoregulatory factor, vitamin D deficiency leads to dysbiosis of gut microbiome and cause severe colitis [28–30]. In another aspect, vitamin D supplementation was found to prevent the occurrence of postoperative AF [31]. From the perspective of therapeutic drugs, there were cases reported that several patients with IBD had incidences of AF after taking azathioprine, indicating that medication may be another potential confounder for the association between IBD and AF [32, 33].

The null association found in our study could be explained by several interpretations. First, there may not be sufficient power to support a significant association between IBD and AF. Our MR analysis has 80% power to detect small effect sizes for the development of AF. However, with our tight confidence intervals, a very small effect of IBD on AF could not be excluded. Second, IBD susceptibility variants tended to be associated with both higher and lower risk of AF, which may cancel the causal effect, resulting in a null association. However, leave-one-out sensitivity analysis showed that the null association was not remarkably affected by any individual SNP. Third, some individuals in the datasets for IBD may have been taking medication, which may distort the relationship between IBD and AF.

Given that heterogeneous allele frequencies in more diverse populations may lead to bias in the genetic study, it is better to restrict MR analyses to homogeneous ethnic populations [34]. As reported in the GWAS summary datasets, cases and controls were of European ancestry. Principal components analysis was performed to resolve geographic stratification, as well as Jewish and non-Jewish ancestry [18]. The effect of bias is expected to be negligible given the population has the same ethnic backgrounds. Also while removing participants with arrhythmia from the analyses to avoid assessment bias, the AF polygenic risk score was no longer correlated with heart valve disorders, heart failure, and ischemic heart disease, suggesting that these additional associations were mediated through AF.

Compared to traditional observational studies, MR analysis is less prone to potential confounding and avoids reverse causation because genetic variation is allocated at conception, and thus, it can strengthen the evidence for causal inference. The null association may be subjected to potential biases if key assumptions are violated. However, the estimation of a false negative effect is attributed to a complicated pattern in the distribution of biases. As these biases rarely neutralize each other, there is a higher chance to estimate a false positive effect than a false negative one. Considering these asymmetry-related biases, the null association found in this study may provide more robust evidence to support no or very little effect of IBD on AF. This investigation on AF susceptibility provides a broad perspective of the relationship between IBD and the development of AF. The clinical applicability of our study is mainly from a therapeutic perspective. Given the null association found in this study, large uncertainty about the potential therapeutic benefits was raised, suggesting that the intention to decrease AF or AF towards the disease (such as stroke) using the treatment for IBD may unlikely be successful and should not be prioritized in future trials.

Our study has several limitations. First, it is unlikely to remove all potential horizontal pleiotropy from the study, which may result in biased estimation of causal inference [35], however, no pleiotropic effect was detected in the MR-Egger regression, MR-PRESSO, or heterogeneity test. Second, given the necessity of identical gene-exposure associations across datasets, two-sample MR analysis should be sufficiently homogeneous, which may be violated in practice [36]. Third, causal effect size may not be treated reliably as it is biased when a causal effect does exist, but the bias decreases as the causal estimate tend to unity. In this investigation, a "null" causal effect was obtained; therefore the bias should not be large. Fourth, there is no easy way to assess the instrument strength of each SNP and statistical power to obtain casual association estimates of a certain size using binary exposures.

## CONCLUSIONS

In this study, two-sample MR analysis did not provide convincing evidence to support a causal effect of IBD on the risk of AF.

## MATERIALS AND METHODS

### Study design and data sources

**Figure 5:**
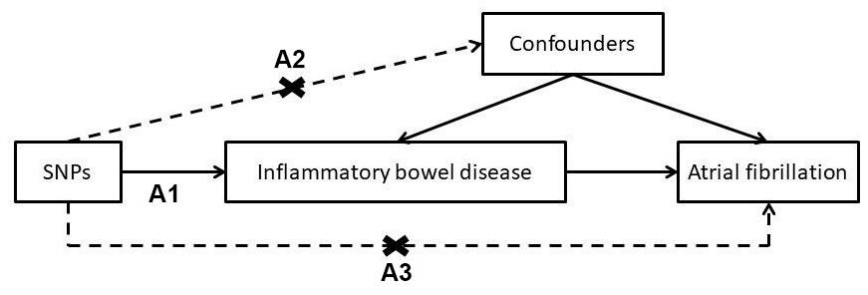
**Conceptual framework for the mendelian randomization analysis of inflammatory bowel disease and risk of atrial fibrillation.** (**A1**) genetic variants are associated with the risk factor; (**A2**) genetic variants are not associated with any confounder of the association between the risk factor and outcome; and (**A3**) genetic variants are not associated with the outcome conditional on the risk factors and confounders.

A two-sample MR approach was used to investigate the causal effect of IBD on AF (Figure 5), using summary-level data from public genome-wide association studies (GWAS). Details of the dataset used to evaluate the association between genetic variants, IBD and AF are listed in Table 1. Given that genetic variants may exhibit different pleiotropic effects in trans-ancestry cases, estimated associations derived from individuals with the same ancestry may prevent the bias of population admixture [37]. The dataset summary was acquired from GWAS [38] which allows secondary analysis through copying and redistributing the material in any medium or format [39]. Since the analysis was based on the dataset of a published study containing no personal identifications, no ethical approval was required.

**Table 1:** Description of contributing studies.

**Contribution**	**Trait**	**Sample size**	**Number of SNPs**	**Author**	**PMID**	**Population**
Exposure	Inflammatory bowel disease	76,312	14,378	Jostins L	23128233	European
Outcome	Atrial fibrillation	1,030,836	33,519,037	Nielsen JB	30061737	European

### Selection of genetic variants

Summary statistics for SNPs related to IBD were acquired from public GWAS summary datasets. The individuals in this dataset were of European ancestry. More than 75,000 cases and controls were included to identify potential IBD loci that met genome-wide significance thresholds [18]. The p-value threshold used for instrument selection was 5x10^-8^. LD proxies were defined using 1000 genomes European sample data. To avoid double counting of the effects of a particular causal variant, strict linkage disequilibrium (LD) thresholds were used. A minor allele frequency (MAF) threshold of 0.3 for palindromic SNPs was allowed. The clumping threshold was specified as r^2^ < 0.001 over a 10kb region, resulting in 112 extracted instruments. Phenoscanner [40] was used to exclude SNPs (n = 28) which may affect outcome with multiple traits at genome-wide significance level of p < 5.0×10^−8^: 13 SNPs (rs1050152, rs10516487, rs10781499, rs13387729, rs2412970, rs2488389, rs2950835, rs3197999, rs35675666, rs4072037, rs7657746, rs798502, and rs8062405) were associated with body mass index and fat-free mass; 8 SNPs (rs108499, rs1363907, rs907611, rs2382817, rs516246, rs6062504, rs17293632, and rs6673002) were associated with heart rate, blood pressure, and coronary artery disease; 6 SNPs (rs1893217, rs3024505, rs6908425, rs6927022, rs7911264, and rs9170) were associated with diabetes; and 1 SNP (rs12654812) was associated with chronic kidney disease. Also, using MR pleiotropy residual sum and outlier (MR-PRESSO), three SNPs (rs10800309, rs2266959, and rs12946510) were identified as horizontal pleiotropic outliers and removed. The characteristics and associations with IBD of the remaining 81 SNPs used as instruments in the MR analysis are shown in Supplementary Table 2. The instrumental variable explained 9.0% of the variance in the liability of the IBD exposure.

### Outcomes

Summary statistics for the 81 SNPs related to AF were acquired from public GWAS summary datasets. The dataset was from six contributing studies, including 1,030,836 individuals of European ancestry (60,620 AF cases vs. 970,216 controls) [19]. Cases with AF were identified by the International Classification of Diseases (Tenth Revision, ICD-10: I48; Ninth Revision, ICD-9 code 427.3).

### Statistical analysis

A two-sample MR approach was employed to estimate the causal effect of IBD on AF using summarized data of the SNP-AF and SNP-IBD associations. Using SNPs as instrumental variables (IV), MR analysis rests on the following three key assumptions in this study: The first assumption is that the SNPs must be associated with exposure (IBD). The second assumption is that the SNP should not be associated with confounders of the association between risk factors and outcomes. The third assumption is that the SNPs should affect the outcome (AF) only through the risk factor (IBD). Based on these assumptions, unconfounded associations can be estimated by controlling for potential confounders and reverse causation. Multiplicative random-effects inverse-variance weighted (IVW) MR analyses were performed. The effect estimate was set as the IVW mean of ratio estimates from two or more instruments using first-order weights [41]. To limit the bias of pleiotropy effects [42], a sensitivity analysis was conducted with MR-Egger, simple median, weighted median, robust adjusted profile score (RAPS) [43], and MR-PRESSO [35] methods of MR analyses. The intercept test for MR-Egger was used to detect “directional” pleiotropic effects [43]. Despite a weaker power to avoid detecting a null association, the MR-Egger method provides a more robust estimate of potential violations of the standard instrumental variable assumptions in the presence of directional pleiotropy [44]. The weighted median approach allows the IV assumptions to be violated in a more general way. The weighted median estimator for combining data on multiple genetic variants into a single causal estimate is consistent even when up to 50% of the information comes from invalid instrumental variables [45]. The RAPS proposed a consistent and asymptotically normal estimator by adjusting the profile score [46]. The idiosyncratic pleiotropy was tackled by robustifying the adjusted profile score. Using MR-PRESSO [35], horizontal pleiotropic outliers were identified and a pleiotropy-corrected estimate was reported. Heterogeneity between SNPs in the IVW analysis was assessed using the Q statistic and I^2^ index [47]. In addition, a leave-one-out analysis was performed to evaluate the influence of outlying or pleiotropic SNPs [48]. The associations between genetically predicted IBD and AF were presented as log odds ratios (OR) with their 95% confidence intervals (CIs) per unit increase in the log OR of IBD [49]. Using a web-based application (http://cnsgenomics.com/shiny/mRnd/), with a sample size of 76,312, MR analysis has 80% power at an alpha rate of 5% to detect an OR of 1.07 per log odds of IBD. The alpha level for statistical significance for causal effects was defined as less than 0.05 (two-sided). Data were processed using STATA software version 16 (StataCorp, TX, USA) and R 3.2.5 (R Development Core Team). MR-PRESSO is a bootstrap method dependent on random number generation (RNG). The RNG seed used in R was set at 6.

### Data availability statements

The dataset that would be necessary to interpret, replicate were provided [18, 19].

### Availability of data and material

Included in ‘Method’ section.

## Supplementary Material

Supplementary Tables
