# Supplementary material for: Mendelian randomization as an approach to assess causal effects of inflammatory bowel disease on atrial fibrillation
Source: Aging (Albany NY). 2021 Apr 6;13(8):12016–30. doi: 10.18632/aging.202906 (PMC8109086; doi:10.18632/aging.202906)
Supplement: Supplementary Tables [file aging-13-202906-s001.pdf]

SUPPLEMENTARY TABLES

Supplementary Table 1. Causal estimate with MR-egger and multiplicative random effects IVW (each SNP and overall effect).

SNP	Beta	SE	p-value
rs1042058	-0.0775	0.0958	0.4186
rs10521318	-0.0424	0.0896	0.6357
rs1062158	-0.1723	0.0843	0.0409
rs10758669	-0.0217	0.0471	0.6451
rs10761659	-0.0121	0.0428	0.7767
rs11209026	-0.0059	0.0196	0.7625
rs11230563	0.0496	0.0846	0.5581
rs11564126	0.0260	0.0743	0.7260
rs11597483	-0.1413	0.0622	0.0231
rs11672983	0.0488	0.1005	0.6275
rs11742570	0.0267	0.0371	0.4712
rs11879191	0.0545	0.0693	0.4320
rs12142199	-0.0880	0.0880	0.3173
rs1250550	-0.1594	0.0724	0.0278
rs12722515	0.1418	0.0964	0.1412
rs1292053	-0.2752	0.0960	0.0042
rs12942547	-0.0648	0.0630	0.3033
rs13009506	0.0536	0.0484	0.2687
rs13019081	-0.0509	0.0707	0.4712
rs1456896	-0.0371	0.0798	0.6421
rs1516976	0.0333	0.1019	0.7440
rs1517352	-0.0387	0.0907	0.6698
rs1558744	0.0850	0.0615	0.1669
rs1569723	0.1652	0.0949	0.0818
rs17085007	0.1221	0.0830	0.1412
rs17119	-0.0225	0.0909	0.8049
rs17396847	-0.1128	0.0786	0.1513
rs17694108	-0.0933	0.0764	0.2222
rs1847472	0.0227	0.0994	0.8192
rs1872691	0.0477	0.0676	0.4803
rs2024092	-0.0009	0.0753	0.9903
rs2155219	0.0850	0.0482	0.0782
rs2413583	-0.1309	0.0501	0.0089
rs243323	0.1358	0.0770	0.0778
rs259964	0.0469	0.0850	0.5808
rs2816954	-0.2728	0.0973	0.0051
rs2823286	-0.0355	0.0525	0.4992
rs2836878	0.0813	0.0424	0.0549
rs2930047	-0.1093	0.1062	0.3035
rs3091316	0.0150	0.0644	0.8159
rs3764147	0.0173	0.0866	0.8415

rs3851228	-0.0252	0.0857	0.7687
rs4246905	0.0890	0.0506	0.0784
rs4380874	-0.1229	0.0731	0.0927
rs4409764	-0.0169	0.0385	0.6604
rs4626115	-0.0532	0.0893	0.5513
rs4656958	0.1620	0.1027	0.1147
rs4743820	0.1519	0.1036	0.1427
rs4921227	-0.0224	0.0481	0.6421
rs559928	0.1718	0.0886	0.0523
rs6017342	0.0067	0.0585	0.9090
rs6142618	-0.0235	0.0997	0.8140
rs6426833	0.1137	0.0540	0.0352
rs6588248	-0.0338	0.0798	0.6714
rs6592362	-0.0312	0.1093	0.7751
rs6724516	-0.0978	0.0978	0.3173
rs6740462	0.0383	0.1079	0.7224
rs683078	-0.0091	0.1018	0.9286
rs6879495	0.0796	0.0847	0.3475
rs6911490	-0.0343	0.0686	0.6171
rs6920220	0.1063	0.0793	0.1798
rs7097656	-0.1681	0.0784	0.0320
rs7200798	0.0651	0.1064	0.5406
rs7214193	0.2440	0.1105	0.0273
rs7240004	-0.0165	0.1034	0.8733
rs727088	-0.0535	0.1025	0.6014
rs7495132	-0.0468	0.0886	0.5973
rs7554511	-0.0538	0.0498	0.2797
rs7608910	0.0663	0.0526	0.2074
rs762422	-0.1105	0.0637	0.0830
rs7724036	-0.0501	0.0594	0.3992
rs7749278	0.0873	0.0749	0.2433
rs7788750	0.0356	0.1049	0.7345
rs8005161	0.0828	0.0779	0.2883
rs8107703	-0.1603	0.1011	0.1129
rs913678	0.0601	0.1110	0.5880
rs921720	-0.0804	0.0770	0.2964
rs941823	0.0062	0.0957	0.9482
rs9557195	-0.0318	0.0804	0.6928
All - IVW (multiplicative random effects)	-0.0028	0.0090	0.7527
All - MR Egger	-0.0002	0.0210	0.9913
MR-PRESSO	-0.0028	0.0090	0.7550

Abbreviation: MR, Mendelian Randomization; IVW, Inverse-Variance Weighted; SE, Standard Error; SNPs , Single Nucleotide Polymorphisms.

Supplementary Table 2. Characteristics of the 81 SNPs related to atrial fibrillation and inflammatory bowel disease.

SNP	Effects on inflammatory bowel disease						Effects on atrial fibrillation					
	EA	OA	EAF	Beta	SE	p-val	EA	OA	EAF	Beta	SE	p-val
rs1042058	C	T	0.5920	0.0710	0.0112	2.0900×10^{-10}	C	T	0.5930	-0.0055	0.0068	0.4176
rs10521318	T	C	0.0846	-0.1273	0.0210	1.4100×10^{-9}	T	C	0.0982	0.0054	0.0114	0.6323
rs1062158	T	C	0.6290	0.0807	0.0114	1.2100×10^{-12}	T	C	0.6156	-0.0139	0.0068	0.0420
rs10758669	A	C	0.6510	-0.1613	0.0115	7.8800×10^{-45}	A	C	0.6473	0.0035	0.0076	0.6418
rs10761659	G	A	0.5430	0.1565	0.0111	8.4700×10^{-45}	G	A	0.5433	-0.0019	0.0067	0.7766
rs11209026	A	G	0.0670	-0.7095	0.0263	8.1300×10^{-161}	A	G	0.0625	0.0042	0.0139	0.7626
rs11230563	T	C	0.3460	-0.0827	0.0116	9.0300×10^{-13}	T	C	0.3456	-0.0041	0.0070	0.5601
rs11564126	G	A	0.0310	0.3418	0.0342	1.6700×10^{-23}	G	A	0.0187	0.0089	0.0254	0.7269
rs11597483	G	A	0.3460	0.1125	0.0114	5.2400×10^{-23}	G	A	0.3382	-0.0159	0.0070	0.0229
rs11672983	A	G	0.3920	0.0677	0.0113	2.4800×10^{-9}	A	G	0.3709	0.0033	0.0068	0.6211
rs11742570	C	T	0.6050	0.1834	0.0113	3.4600×10^{-59}	C	T	0.6020	0.0049	0.0068	0.4723
rs11879191	A	G	0.2030	-0.1212	0.0138	2.0400×10^{-18}	A	G	0.1995	-0.0066	0.0084	0.4275
rs12142199	A	G	0.8110	-0.0977	0.0150	7.5900×10^{-11}	A	G	0.7787	0.0086	0.0086	0.3184
rs1250550	A	C	0.3240	-0.0966	0.0121	1.1900×10^{-15}	A	C	0.3596	0.0154	0.0070	0.0281
rs12722515	A	C	0.1510	-0.0903	0.0155	5.8200×10^{-9}	A	C	0.1860	-0.0128	0.0087	0.1413
rs1292053	G	A	0.4460	0.0698	0.0109	1.7700×10^{-10}	G	A	0.4415	-0.0192	0.0067	0.0039
rs12942547	G	A	0.4200	-0.1080	0.0112	5.5100×10^{-22}	G	A	0.4374	0.0070	0.0068	0.3004
rs13009506	T	G	0.5230	0.1363	0.0110	3.4700×10^{-35}	T	G	0.5188	0.0073	0.0066	0.2688
rs13019081	C	A	0.3750	-0.0962	0.0113	3.0000×10^{-17}	C	A	0.3864	0.0049	0.0068	0.4712
rs1456896	T	C	0.6880	0.0889	0.0119	9.7500×10^{-14}	T	C	0.6607	-0.0033	0.0071	0.6444
rs1516976	C	T	0.1340	-0.0962	0.0166	5.7700×10^{-9}	C	T	0.1345	-0.0032	0.0098	0.7434
rs1517352	C	A	0.6000	0.0749	0.0113	3.2800×10^{-11}	C	A	0.6094	-0.0029	0.0068	0.6695
rs1558744	A	G	0.3990	0.1106	0.0112	2.6300×10^{-23}	A	G	0.4146	0.0094	0.0068	0.1641
rs1569723	A	C	0.7410	-0.0811	0.0124	6.5500×10^{-11}	A	C	0.7363	-0.0134	0.0077	0.0822
rs17085007	C	T	0.1830	0.1048	0.0144	3.7500×10^{-13}	C	T	0.1761	0.0128	0.0087	0.1422
rs17119	A	G	0.7860	0.0935	0.0141	3.0700×10^{-11}	A	G	0.7892	-0.0021	0.0085	0.8075
rs17396847	A	G	0.3850	-0.0877	0.0119	1.6900×10^{-13}	A	G	0.3888	0.0099	0.0069	0.1523
rs17694108	A	G	0.2820	0.1008	0.0129	5.8500×10^{-15}	A	G	0.2952	-0.0094	0.0077	0.2208
rs17835641	C	G	0.5230	-0.0683	0.0110	4.8800×10^{-10}	C	G	0.5058	-0.0085	0.0066	0.2024
rs1847472	A	C	0.3450	-0.0704	0.0116	1.1000×10^{-9}	A	C	0.3485	-0.0016	0.0070	0.8197
rs1872691	A	G	0.1800	-0.1258	0.0146	5.5900×10^{-18}	A	G	0.1860	-0.0060	0.0085	0.4801
rs1991866	C	G	0.5780	-0.0672	0.0112	1.6500×10^{-9}	C	G	0.5768	0.0092	0.0068	0.1735
rs2024092	A	G	0.2150	0.1089	0.0136	1.2300×10^{-15}	A	G	0.2046	-0.0001	0.0082	0.9923
rs2155219	T	G	0.5090	0.1389	0.0111	4.2400×10^{-36}	T	G	0.4977	0.0118	0.0067	0.0753
rs2413583	T	C	0.1670	-0.1818	0.0152	4.4000×10^{-33}	T	C	0.1590	0.0238	0.0091	0.0090
rs243323	G	A	0.3110	-0.0935	0.0120	6.1200×10^{-15}	G	A	0.2911	-0.0127	0.0072	0.0800
rs259964	G	A	0.5360	-0.0788	0.0111	1.0100×10^{-12}	G	A	0.5428	-0.0037	0.0067	0.5775
rs2816954	A	T	0.8490	0.0935	0.0167	2.4000×10^{-8}	A	T	0.8248	-0.0255	0.0091	0.0052
rs2823286	A	G	0.2920	-0.1410	0.0124	9.2800×10^{-30}	A	G	0.2920	0.0050	0.0074	0.4999
rs2836878	A	G	0.2670	-0.1771	0.0129	7.2700×10^{-43}	A	G	0.2795	-0.0144	0.0075	0.0551
rs2930047	C	T	0.3820	0.0650	0.0113	1.0300×10^{-8}	C	T	0.3599	-0.0071	0.0069	0.3006
rs3091316	A	G	0.2780	-0.1134	0.0124	5.2600×10^{-20}	A	G	0.2947	-0.0017	0.0073	0.8131

rs3764147	G	A	0.2480	0.0923	0.0128	5.6300×10^{-13}	G	A	0.2116	0.0016	0.0080	0.8410
rs3851228	T	A	0.0730	0.1586	0.0213	1.0800×10^{-13}	T	A	0.0646	-0.0040	0.0136	0.7694
rs4246905	C	T	0.7090	0.1483	0.0125	2.8000×10^{-32}	C	T	0.7063	0.0132	0.0075	0.0771
rs4380874	C	T	0.5950	-0.0944	0.0113	7.1300×10^{-17}	C	T	0.5863	0.0116	0.0069	0.0925
rs4409764	G	T	0.5090	-0.1714	0.0110	1.0300×10^{-54}	G	T	0.5111	0.0029	0.0066	0.6653
rs4626115	T	C	0.1380	0.1053	0.0155	1.1000×10^{-11}	T	C	0.1471	-0.0056	0.0094	0.5536
rs4656958	G	A	0.6860	0.0691	0.0119	6.8000×10^{-9}	G	A	0.6692	0.0112	0.0071	0.1152
rs4743820	T	C	0.7020	0.0705	0.0122	6.4500×10^{-9}	T	C	0.6987	0.0107	0.0073	0.1444
rs4921227	G	A	0.3290	0.1476	0.0116	6.1900×10^{-37}	G	A	0.3332	-0.0033	0.0071	0.6473
rs559928	C	T	0.8210	0.0949	0.0144	4.1900×10^{-11}	C	T	0.7930	0.0163	0.0084	0.0528
rs6017342	C	A	0.5300	0.1196	0.0125	1.4300×10^{-21}	C	A	0.5194	0.0008	0.0070	0.9054
rs6142618	G	A	0.5640	0.0682	0.0110	6.0500×10^{-10}	G	A	0.5726	-0.0016	0.0068	0.8142
rs6426833	A	G	0.5420	0.1222	0.0110	2.0200×10^{-28}	A	G	0.5181	0.0139	0.0066	0.0359
rs6588248	G	T	0.5370	0.0827	0.0110	4.9800×10^{-14}	G	T	0.5250	-0.0028	0.0066	0.6768
rs6592362	G	A	0.7520	-0.0705	0.0126	2.3200×10^{-8}	G	A	0.7281	0.0022	0.0077	0.7697
rs6724516	A	G	0.7200	0.0797	0.0126	2.2300×10^{-10}	A	G	0.7400	-0.0078	0.0078	0.3209
rs6740462	A	C	0.7390	0.0705	0.0126	2.3500×10^{-8}	A	C	0.7245	0.0027	0.0076	0.7198
rs683078	C	T	0.4830	-0.0658	0.0110	2.4300×10^{-9}	C	T	0.4458	0.0006	0.0067	0.9300
rs6879495	T	C	0.5310	0.0779	0.0111	2.4600×10^{-12}	T	C	0.5172	0.0062	0.0066	0.3498
rs6911490	C	T	0.7920	-0.1196	0.0138	3.8000×10^{-18}	C	T	0.7696	0.0041	0.0082	0.6222
rs6920220	A	G	0.2060	0.1035	0.0134	1.0100×10^{-14}	A	G	0.2107	0.0110	0.0082	0.1770
rs7097656	C	T	0.8020	0.1059	0.0139	2.0500×10^{-14}	C	T	0.7826	-0.0178	0.0083	0.0329
rs7200798	A	G	0.5270	0.0630	0.0110	1.2700×10^{-8}	A	G	0.5493	0.0041	0.0067	0.5448
rs7214193	G	A	0.2230	-0.0742	0.0132	1.6000×10^{-8}	G	A	0.2062	-0.0181	0.0082	0.0277
rs7240004	G	A	0.3840	-0.0667	0.0116	9.8100×10^{-9}	G	A	0.3794	0.0011	0.0069	0.8758
rs727088	A	G	0.5160	-0.0654	0.0112	4.6500×10^{-9}	A	G	0.5122	0.0035	0.0067	0.6051
rs7495132	T	C	0.1090	-0.1196	0.0185	9.4800×10^{-11}	T	C	0.1125	0.0056	0.0106	0.5987
rs7554511	A	C	0.2750	-0.1487	0.0125	1.2400×10^{-32}	A	C	0.2957	0.0080	0.0074	0.2757
rs7608910	G	A	0.3940	0.1312	0.0112	8.6500×10^{-32}	G	A	0.3655	0.0087	0.0069	0.2064
rs762422	A	G	0.6160	-0.1177	0.0112	6.5200×10^{-26}	A	G	0.6072	0.0130	0.0075	0.0818
rs7724036	T	C	0.0944	0.2038	0.0190	1.0300×10^{-26}	T	C	0.0786	-0.0102	0.0121	0.3971
rs7749278	C	T	0.4680	0.0882	0.0110	8.4500×10^{-16}	C	T	0.4791	0.0077	0.0066	0.2439
rs7788750	T	C	0.0459	0.1630	0.0261	4.4400×10^{-10}	T	C	0.0397	0.0058	0.0171	0.7342
rs8005161	T	C	0.0887	0.1450	0.0190	2.3500×10^{-14}	T	C	0.0907	0.0120	0.0113	0.2894
rs8107703	A	G	0.9460	-0.1622	0.0252	1.1900×10^{-10}	A	G	0.9233	0.0260	0.0164	0.1135
rs913678	C	T	0.3380	-0.0649	0.0118	4.5900×10^{-8}	C	T	0.3322	-0.0039	0.0072	0.5932
rs921720	G	A	0.6090	0.0883	0.0113	6.7100×10^{-15}	G	A	0.6170	-0.0071	0.0068	0.2985
rs941823	C	T	0.7580	0.0805	0.0128	3.8400×10^{-10}	C	T	0.7326	0.0005	0.0077	0.9530
rs9557195	C	T	0.2280	-0.1008	0.0133	2.3700×10^{-14}	C	T	0.2095	0.0032	0.0081	0.6936

Abbreviation: EA, Effect Allele; OA, Other Allele; EAF, effect allele frequency; SE, standard error; SNP, single nucleotide polymorphism.
